# Whole-Genome Sequences of Five Oligotrophic Bacteria Isolated from Deep within Lechuguilla Cave, New Mexico

**DOI:** 10.1128/genomeA.01133-14

**Published:** 2014-11-06

**Authors:** Huan You Gan, Han Ming Gan, Alexander Mario Tarasco, Nurfatini Idayu Busairi, Hazel A. Barton, André O. Hudson, Michael A. Savka

**Affiliations:** a School of Science, Monash University Malaysia, Jalan Lagoon Selatan, Bandar Sunway, Petaling Jaya, Selangor, Malaysia; b The Thomas H. Gosnell School of Life Sciences, Rochester Institute of Technology, Rochester, New York, USA; c Department of Biology, University of Akron, Akron, Ohio, USA

## Abstract

Here, we report the whole-genome sequences and annotation of five oligotrophic bacteria from two sites within the Lechuguilla Cave in the Carlsbad Caverns National Park, NM. Three of the five genomes contain an acyl-homoserine lactone signal synthase ortholog (*luxI*) that is involved in cell-to-cell communication via quorum sensing.

## GENOME ANNOUNCEMENT

Bacterial diversity from extreme oligotrophic environments, such as caves, is of interest given the unique substrate environment. As such, we embarked on a project to isolate and identify bacteria from two sites in the Lechuguilla Cave, NM (1). The genomes of bacterial species from caves as deep as Lechuguilla Cave (>400 m) have not yet been sequenced, with only three cave bacterial genomes sequenced to date: *Beutenbergia cavernae* from Guangxi, China, *Pseudomonas fluorescens* from the Guiana Shield, South America, and *Gloeobacter kilaueensis* from a biofilm in Kīlauea Caldera, Hawaii (2–4). The bacterial genomes presented here were isolated from cave surfaces: strain LC238 was collected from an iron- and manganese-rich corrosion residue at the contact between the limestone bedrock and a back reef sandstone (the Yates Formation) at −200 m, while LC5, LC81, LC85, and LC363 were collected from a deep and remote location within the limestone Capitan Formation at −347 m. Each site is extremely nutrient limited, with organic carbon present at between 0.1 and 0.7 mg/g of sediment and with a C-to-N ratio approaching 300:1 (1). The bacteria were initially identified by nucleotide sequence analysis of the variable regions 3 and 4 of the 16S rRNA gene (1), with LC5 and LC363 identified as members of the genera *Devosia* and *Sphingopyxis*, respectively (Table 1).

**TABLE 1:** Sequencing and annotation results of the five cave bacteria isolated from two sites within the Lechuguilla Cave in the Carlsbad Caverns National Park, NM^*a*^

Strain	Source (depth in m)	BioProject no.	Biosample no.	Accession no.	Organism	Genome coverage (×)	Genome size (bp)	No. of contigs	No. of ORFs	No. of tRNAs	No. of rRNAs
LC5	LCEAE (−347)	PRJNA248423	SAMN02798393	JNNO00000000	*Devosia* sp.	65	4,202,991	47	4,117	48	6
LC81	LCEAE (−347)	PRJNA248597	SAMN02799681	JNFD00000000	*Sphingopyxis* sp.	104	4,397,290	48	4,111	44	3
LC85	LCEAE (−347)	PRJNA248600	SAMN02799685	JPKG00000000	*Bosea* sp.	47	6,564,029	74	6,193	51	3
LC238	LCAE1 (−200)	PRJNA248601	SAMN02799686	JNNN00000000	*Massilia* sp.	29	5,799,774	83	5,102	65	7
LC363	LCEAE (−347)	PRJNA248602	SAMN02799687	JNFC00000000	*Sphingopyxis* sp.	28	4,210,757	73	3,908	48	3

a Sites where samples were obtained in the Lechuguilla Cave, Carlsbad Caverns National Park, New Mexico (refer to Fig. 1, profile map of the Lechuguilla Cave system, in reference 1).

The sequencing libraries were prepared from extracted DNA using the Nextera XT kit (Illumina, San Diego, CA). Each sample was tagged with unique bar codes, according to the manufacturer’s protocol. The final libraries were normalized based on the 2100 Bioanalyzer readings (Agilent Technologies) and pooled for sequencing on the MiSeq sequencing system (Illumina) at the Monash University Malaysia Genomics Facility to generate FASTQ files. Raw FASTQ reads for each library were corrected for errors and *de novo* assembled into contigs with the SPAdes Genome Assembler (version 2.5.1) (5). Scaffolds were then generated from the assembled contigs using SSPACE (version 2.0) (6). The gaps in the resulting scaffolds were then closed using GapFiller (version 1.11) (7). The annotation for each genome was performed using the Prokka (version 1.8) annotation pipeline (8), which comprises Aragorn (version 1.2.36), Prodigal (version 2.60), and RNAmmer (version 1.2), which predicted tRNAs, open reading frames (ORFs), and rRNAs, respectively (9–11). The predicted 16S rRNA from RNAmmer was queried using BLASTn against the NCBI nt database. The genus of each sample sequence was determined by manually observing the distance tree of the result to check if the query sequence falls within a certain cluster of organisms (12). InterProScan 5 was used to provide additional annotation to the predicted protein sequences (13). A summary of the key properties for each genome is shown in Table 1. Phylogenetic analysis of the full-length 16S rRNA gene reclassified strain LC238 as a *Massilia* species. Further, genome analysis demonstrated that strains LC81, LC363, and LC238 contain a *luxI* homolog, implicating that cell-to-cell communication is potentially mediated by acyl-homoserine lactones in cave environments.

### Nucleotide sequence accession numbers.

The nucleotide sequences have been deposited at DDBJ/EMBL/GenBank under the accession numbers provided in Table 1.
